# Shallow and deep trap states of solvated electrons in methanol and their formation, electronic excitation, and relaxation dynamics†

**DOI:** 10.1039/d1sc06666h

**Published:** 2022-03-11

**Authors:** Jinggang Lan, Yo-ichi Yamamoto, Toshinori Suzuki, Vladimir V. Rybkin

**Affiliations:** Department of Chemistry, University of Zurich, Winterthurerstrasse 190, Zurich 8057 Switzerland jinggang.lan@epfl.ch vladimir.rybkin@quantumsimulations.de; Department of Chemistry, Graduate School of Science, Kyoto University Kyoto 606-8502 Japan suzuki@kuchem.kyoto-u.ac.jp

## Abstract

We present condensed-phase first-principles molecular dynamics simulations to elucidate the presence of different electron trapping sites in liquid methanol and their roles in the formation, electronic transitions, and relaxation of solvated electrons (e_met_^−^) in methanol. Excess electrons injected into liquid methanol are most likely trapped by methyl groups, but rapidly diffuse to more stable trapping sites with dangling OH bonds. After localization at the sites with one free OH bond (1OH trapping sites), reorientation of other methanol molecules increases the OH coordination number and the trap depth, and ultimately four OH bonds become coordinated with the excess electrons under thermal conditions. The simulation identified four distinct trapping states with different OH coordination numbers. The simulation results also revealed that electronic transitions of e_met_^−^ are primarily due to charge transfer between electron trapping sites (cavities) formed by OH and methyl groups, and that these transitions differ from hydrogenic electronic transitions involving aqueous solvated electrons (e_aq_^−^). Such charge transfer also explains the alkyl-chain-length dependence of the photoabsorption peak wavelength and the excited-state lifetime of solvated electrons in primary alcohols.

## Abstract 2

Condensed-phase first-principles molecular dynamics simulations elucidate the presence of different electron trapping sites in liquid methanol and their roles in the formation, electronic transitions, and relaxation of solvated electrons.

## Graphical abstract

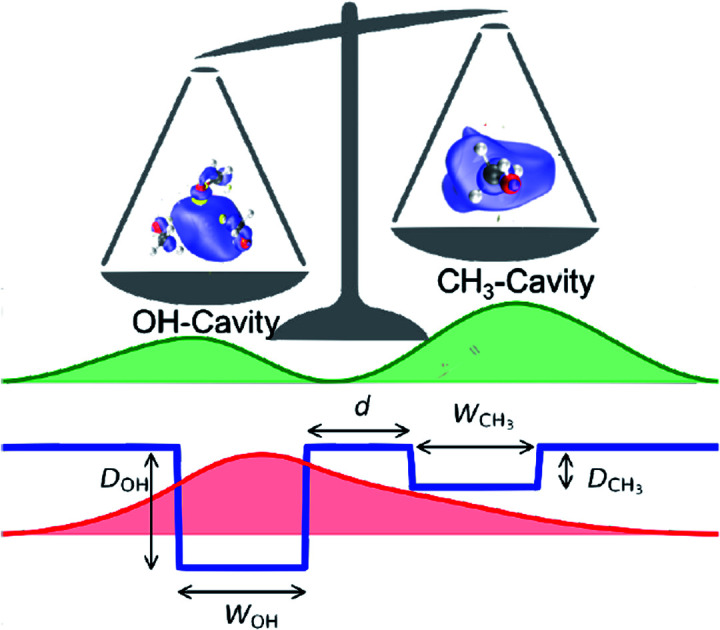

## 1 Introduction

Since solvated electrons (e_sol_^−^) were spectroscopically identified in the 1960s, they have attracted much attention as prototypical free radicals and the most fundamental reducing reagents in solutions.^1,2^ They play crucial roles in ionization, charge transfer, and redox processes in solutions, and also potentially cause radiation damage of biological systems.^3^ However, owing to their short lifetimes and low concentrations, experimental determination of their geometrical structures using electron/X-ray diffraction or nuclear magnetic resonance has been difficult. Consequently, structural information has been obtained indirectly using electronic, vibrational, and electron paramagnetic resonance spectroscopy. In view of these experimental limitations, theoretical studies are expected to be of assistance in elucidating the structure and dynamics of e_sol_^−^, even though theoretical studies are as challenging as experimental ones.^4–7^

So far, most attention has been devoted to aqueous solvated electrons, e_aq_^−^; however, their analogues in other solvents are also highly interesting and valuable for practical applications. e_sol_^−^ in ammonia have been utilized for organic synthesis processes such as Birch reduction.^8^ However, since Birch reduction requires low temperatures and the use of liquid ammonia, efforts have been made to develop alternative reactions using other solvents such as alcohols and ethers.^9,10^ The Bouveault–Blanc reaction employs e_sol_^−^ in alcohols for reducing esters to primary alcohols.^11^ Thus, a detailed study on the structure and dynamics of e_sol_^−^ in alcohols may contribute to developing highly efficient and environmentally benign reduction reactions.^9–11^

Despite apparent similarities, solvated electrons in methanol, e_met_^−^, exhibit some notable differences from e_aq_^−^. Most importantly, two trap states – shallow and deep – have been experimentally identified for e_met_^−^ in the ground electronic state.^12^ The shallow trap state with a near-infrared absorption band was identified in low-temperature (4–77 K) glass, and its transformation to a deep trap state was observed at temperatures above 77 K, based on the emergence of a visible absorption band.^13,14^ The deep trap state has been observed for a liquid methanol microjet using photoemission spectroscopy.^15–17^ A recent femtosecond pump–probe photoemission spectroscopy study of e_met_^−^ (ref. 12) found that the vertical electron binding energy (VBE) for these two trap states is 2.1 eV (shallow) and 3.4 eV (deep), and that the transformation among these trap states takes place within tens of picoseconds under ambient conditions. Such multiple trap states have not been identified for e_sol_^−^ in water or ammonia. It has been suggested that electrons trapped on the water surface and in the bulk have different VBEs of 1.6 and 3.3 eV,^18,19^ respectively, but this claim has not been supported by clear evidence.^20^

Another noteworthy difference between e_aq_^−^ and e_met_^−^ is in their UV-visible absorption spectrum.^21–23^ Although both spectra exhibit a broad peak with a maximum at similar photon energies (1.7 eV for water and 2.0 eV for methanol), e_met_^−^ exhibits a much higher spectral intensity beyond 3.0 eV. Previous theoretical studies indicated that the main absorption band for e_aq_^−^ is due to overlapping electronic transitions from s-like ground state orbitals to three p-like orbitals and ascribed a long “blue tail” to transitions to higher bound/continuum states.^24^ A similar assignment has been proposed for e_met_^−^.^25,26^ However, while the spectra computed so far for e_met_^−^ reproduced the main absorption band, they did not reproduce the intense absorption feature observed beyond 3.0 eV. It should also be noted that the experimental results indicate that the lifetimes of the electronically excited states for e_sol_^−^ in primary alcohols depend on the carbon chain length, hinting at a qualitatively different character for these states from those for e_aq_^−^.^23,27,28^

These intriguing experimental observations deserve detailed theoretical and computational analyses. Some important theoretical studies have already been published for e_met_^−^. For example, Walker and Bartels^29^ performed calculations on negatively charged methanol clusters in a dielectric medium and suggested that the structure of e_met_^−^ in a bulk solution is a cavity state that is tetrahedrally coordinated with four hydroxy groups, similar to e_aq_^−^. This species with a binding energy of 3.5 eV likely corresponds to the deep trap state. Similar structures have been obtained using condensed-phase molecular dynamics (MD) simulations with empirical force fields augmented by a one-electron Hamiltonian for the excess electron.^25,30–32^ These cluster and bulk simulations suggested that the methyl groups of methanol contribute to trapping of excess electrons with a coordination number of 5–7;^25,30,33,34^ however, it has not been clarified how such trapping contributes to the formation, electronic excitation, and relaxation dynamics of e_met_^−^.

Theoretical study of electron dynamics in methanol is a challenging task, requiring reliable and accurate computational methods. The aforementioned force-field one-electron Hamiltonian models provided important insights into excess electrons; however, these computations were critically dependent on multiple parameters, various choices of which led to qualitatively different results. For example, in the case of e_aq_^−^, different parametrizations predicted completely different structures for the cavity and non-cavity states.^35–38^ Similarly, two different model parameters for e_met_^−^ led to different structures with solvation by either methyl or hydroxy groups.^39^ An alternative less unambiguous approach taken in the present study is to use modern many-electron quantum-chemical methods of dispersion-corrected hybrid density functional theory (DFT) and second-order Møller–Plesset perturbation theory (MP2), which have recently become available for large-scale condensed-phase MD. They involve only a few (dispersion-corrected hybrid DFT) or no (MP2) tunable parameters and provide a reliable description of e_sol_^−^ while keeping the pernicious self-interaction error under control.^40^ The accuracy of these computational methods has been demonstrated for electrons^41–43^ and dielectrons in liquid water^44^ and ammonia,^45^ and for the reaction of e_aq_^−^ with carbon dioxide.^46^

In the present study, we attempt to elucidate the nature of electron trap states in liquid methanol. This is achieved by condensed-phase MD simulations driven by dispersion-corrected hybrid DFT and MP2. After injection of an excess electron into neat liquid methanol, we trace its localization in real time from the “birth” of a shallow trap state to its conversion into the deep trap state in several picoseconds. Although this timescale is insufficient to achieve thermalization of e_met_^−^, we can capture and characterize the time-evolution of the trap state. The results illustrate essential differences in the electronic structure and relaxation dynamics between e_met_^−^ and e_el_^−^.

## 2 Computational methods

### 2.1 Molecular dynamics

We performed MD simulations under periodic boundary conditions for two different system sizes of 39 and 55 methanol molecules with a single excess electron. We calculated the temporal evolution of the system (in the microcanonical ensemble) after injection of an electron into neat liquid methanol under ambient conditions. The initial solvent structures were obtained by equilibration in the NVT ensemble at 300 K at a density corresponding to the experimental value.

Relaxation of the excess electron was traced up to 5 ps with 5 trajectories for each system size using dispersion-corrected hybrid DFT. For the smaller system with 39 molecules, two trajectories were computed up to *ca.* 1 ps with MP2 to confirm the validity of the DFT calculations. MP2 calculations for the larger system are unfeasible at the current state of hardware and software. Thus, most of the analysis was performed on the DFT trajectories, unless otherwise specified. Although the experimentally measured relaxation times are much longer than the time scale of our simulations, the latter are still sufficient for capturing the formation of both shallow and deep trap states. More details on the MD simulations are given in the ESI (Section C).†

### 2.2 Electronic structure theory

As described earlier, *ab initio* MD simulations were performed using hybrid DFT and MP2,^47,48^ which previously provided highly accurate computational results for e_aq_^−^.^41,47,49,50^ The functional of choice is PBE(*α*) with 50% exact exchange with a non-local van der Waals correction, rVV10,^51^ using a triple-zeta quality basis set,^52^ which is proven to provide spin densities and dynamics accurately, similar to those for MP2. A correlation-consistent triple-zeta quality basis set was employed for MP2.^53^ We computed the photoabsorption spectra for the frames along the trajectories using time-dependent DFT (TDDFT) calculations with the same functional and basis set. We also performed a calculation based on the density functional embedding theory^54^ for a selected representative structure of e_met_^−^. All periodic calculations were performed using the CP2K program^55^ in the spin-unrestricted formalism for the excess electron using a uniform positive background charge to compensate for the excess electron's negative charge. The calculations of the cluster model were performed using the ORCA program.^56^ More details of the electronic structure calculations are given in the ESI (Sections A (DFT), B (MP2) and D (TDDFT)).†

### 2.3 Binding energy

We estimated the VBE for e_met_^−^ from the (negative) energy of a singly occupied molecular orbital (SOMO) containing the excess electron. However, the difficulty with this approach using MD simulations with periodic boundary conditions is that the calculated SOMO energy, *E*(SOMO), is defined with respect to an arbitrary zero of energy, so that its absolute energy shifts randomly for each snapshot.^49,57^ In order to eliminate this random shift, we calculated the energy of the semi-core oxygen 2s-orbital, *E*(O_2s_), which is expected to be unaffected by the nuclear configuration or the presence of the excess electron, and subtracted *E*(O_2s_) from *E*(SOMO). For calculations of the VBE, the vacuum energy level is a necessary parameter. In our calculations, we simply assumed that the initial electronic energy upon excess electron injection to liquid methanol is equal to the vacuum level; therefore, the VBE calculated for the *i*-th frame of the MD simulation is approximated as the energy difference between the *i*-th frame and the zeroth frame of the snapshot:

1 VBE_*i*_ = *E*(SOMO)_*i*_ − *E*(O_2s_)_*i*_ − (*E*(SOMO)_0_ − *E*(O_2s_)_0_),

where *i* is the index of the snapshot. As described later, VBEs thus computed were found to be systematically smaller than the experimental values by 0.4 eV; therefore, the calculated VBE values were shifted by +0.4 eV for comparison with the experimental values.

## 3 Results and discussion

### 3.1 Structure and dynamics

**Fig. 1:**
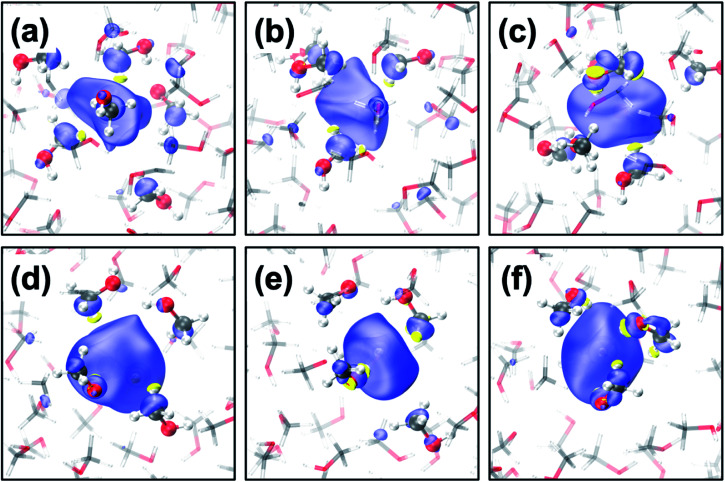
(a–c) Transient diffusion from the CH_3_-cavity to the 1OH-cavity; (d–f) transition from the 2OH-cavity to the 4OH-cavity. Color code: carbon (grey), oxygen (red), hydrogen (white) and positive spin density (blue), negative density (yellow) where the isovalue is 0.001 a.u.

**Fig. 2:**
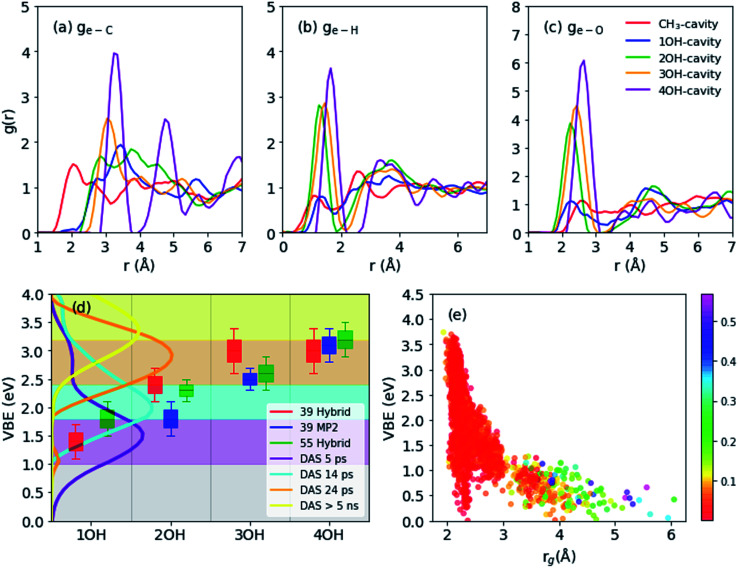
(a–c) Radial distribution functions for main structures (55 molecules): electron distribution center to carbon (a), hydrogen (b), oxygen (c). (d) Vertical electron binding energies (shifted by 0.4 eV to compare with experimental results) of different cavity types as obtained from 39 molecule-system using hybrid functional (red), 39 molecules using MP2 frames (blue), and 55 molecules using hybrid functional frames (green). Decay associated spectra (DAS) calculated by global fitting of photoemission spectra with four-step sequential kinetics model. The spectra are measured by liquid methanol excited by 9.3 eV vacuum UV pulses and probed by 4.3 eV UV pulses (e) vertical binding energies *vs.* spin density gyration radius (55 molecules). The color map corresponds to spin density distribution anisotropy.

The geometrical structure of e_met_^−^ is more complex than that of e_sol_^−^ in water^41,42^ or ammonia^45^ due to the bipolar nature of the solvent with a non-polar methyl- and polar hydroxy-group. Thus, we categorized the cavities into the following five types (see Fig. 1 for the cavities and Fig. 2(a–c) for the radial distribution functions):

(1) CH_3_-cavity: formed by 4–6 methyl groups.

(2) 1OH-cavity: formed by one hydroxyl- and several methyl-groups.

(3) 2OH-cavity: formed by two hydroxyl- and two methyl-groups.

(4) 3OH-cavity: formed by three hydroxyl- and one methyl-group.

(5) 4OH-cavity: formed by four hydroxyl-groups.

CH_3_-cavities occur naturally in liquid methanol because the hydrogen bonding network is one-dimensional. Our simulations indicate that the excess electron is initially localized in a CH_3_-cavity with a compact and relatively isotropic spin-density distribution, in addition to a small VBE, small gyration radius, and weak anisotropy, as shown in Fig. 2(e) (see ESI, Section F†). As soon as one of the hydrogen bonds between solvent molecules breaks to form a dangling OH bond in the vicinity of the CH_3_-cavity, the electron swiftly moves to this polar trap (1OH-cavity). This process is similar in nature to transient diffusion of e_aq_^−^ (see Fig. 1(a–c)).^42^ If a polar trap already exists at the time of electron injection, the electron is most likely trapped immediately in the 1OH-cavity. Thus, at the early stages, the excess electron “looks for” and moves to the most energetically favorable localization site, which may be either a CH_3_- or a 1OH-cavity. This is consistent with the previously discussed concept of the excess electron as “a trap-seeker and not a trap digger”.^12–14^

The dynamics dramatically changes after 2OH-, 3OH- and 4OH-cavities are formed: the solvated electron now behaves as a “trap-digger”. While the electron resides in the same cavity, the methanol molecules initially coordinating their CH_3_-group to the excess electron gradually reorient themselves to coordinate their OH-group. This can occur with one or two molecules at a time (as shown in Fig. 1). For reorientation of a methanol molecule in the first solvation shell, the hydrogen bond between the methanol molecules in the first and second solvation shells must be disrupted. Therefore, the second solvation shell also plays an important role in the solvation dynamics of e_met_^−^, which is reminiscent of the reaction of e_aq_^−^ with CO_2_.^46^ The entire localization process generally follows the following scheme:CH_3_-cavity → 1OH-cavity → 2OH-cavity → 3OH-cavity → 4OH-cavity

However, some of these stages may be completely bypassed or rapidly passed through.

In simulations of the smaller system, three trajectories reached the 4OH-cavity, one 3OH-cavity, and one 2OH-cavity in 5 ps. In the two trajectory calculations at the MP2 level for the smaller system, one trajectory evolved from the CH_3_-cavity to the 2OH-cavity *via* the 1OH-intermediate, and the other transformed from a 3OH-cavity to a 4OH-cavity by reorientation of a methanol molecule: the trajectories at the MP2 level are qualitatively consistent with the results of hybrid DFT calculations. On the other hand, DFT calculations of the larger system indicated that three trajectories reached the 3OH-cavity and two trajectories reached the 2OH-cavity within 5 ps, but no trajectory reached the most stable 4OH-cavity. This is qualitatively consistent with the experimentally measured formation time (tens of picoseconds) for the deep trap state.^12^ However, due to restricted statistics any quantitative theoretical estimates of the transformation time from the shallow are beyond the scope of the present study. We computed the properties of the 4OH-cavity in the larger system by creating its geometrical structure as the initial configuration and thermally relaxing it using MD simulations.

The geometrical structure of e_met_^−^ can be examined in more detail using the radial distribution function (RDF: *g*_(e–X)_ with X = C, H or O) for C, H, and O atoms with respect to the center of the spin density distribution as shown in Fig. 2(a–c). We generally observe that the structure becomes more ordered with increasing OH coordination number even in the case of *g*_(e–C)_, for which the first peak gradually shifts to a larger distance and becomes sharper. For *g*_(e–H)_, the CH_3_- and 1OH-cavities have very similar distributions. The lower-coordinated cavities tend to have shorter distances between the center of the spin-density distribution and the dangling OH-bond, as seen for the *g*_(e–O)_ and *g*_(e–H)_ distributions.

### 3.2 Binding energy

Binding energies calculated for different cavity types are shown in Fig. 2(d): the VBE values obtained for different trajectories generally agree within the error margins. Since the VBE values obtained using eqn (1) were systematically lower than the experimental values by 0.4 eV, the calculated values are shifted by +0.4 eV in Fig. 2(d) for close comparison with the experimental values. This also implies that the initial state (see Section 2.3 and eqn (1)) in our simulation was actually weakly bound (*ca.* 0.4 eV), possibly with an excess electron in the conduction band of liquid methanol, with respect to the vacuum level. This interpretation is qualitatively supported by calculations of an electron binding energy for a methanol cluster (see ESI, Section G†). We note that our simulations identify four rather than two (shallow and deep) trap states with distinct VBE values that are strongly dependent on the OH coordination number. The results indicate that the VBE gradually increases with increasing OH coordination number.

These computational results encouraged us to reinterpret the experimental results reported by Hara *et al.*,^12^ who analyzed the results assuming only two trap states. They performed ultrafast photoemission spectroscopy of liquid methanol with 9.3 eV vacuum UV pulses and examined the subsequent formation of solvated electrons on a picosecond timescale. Based on the VBE values, they classified the observed spectrum of e_met_^−^ to be associated with shallow or deep trap states. These two trap states, however, also exhibited a VBE that increased with time due to solvation (time constants of 15 and 50 ps, respectively). Therefore, neither of these trap states was stationary. The time-evolution of the observed VBE distribution was also analyzed by global fitting, in which the time-dependent photoemission spectra were fit using linear combinations of time-independent spectra of several transient species with different lifetimes (these spectra are called decay associated spectra: DAS). In this analysis scheme, sequential formation of four different species was necessary in order to explain the experimental data. The decay times for these four species were determined to be 5.1 ps, 14 ps, 24 ps, and >5 ns. The VBE distributions for these four states, not presented in the original paper by Hara *et al.*,^12^ are shown in Fig. 2(d). The four states have average VBE values of 1.7, 2.2, 2.8, and 3.3 eV, which are almost equally spaced. Close examination of Fig. 2(d) reveals that the first species has a rather wide VBE distribution and its high-energy tail exceeds 3.0 eV. This implies that some of the electrons are directly trapped in the region where multiple dangling OH-bonds stabilize the electrons, which agrees with our simulation results.

### 3.3 Electronic spectra

**Fig. 3:**
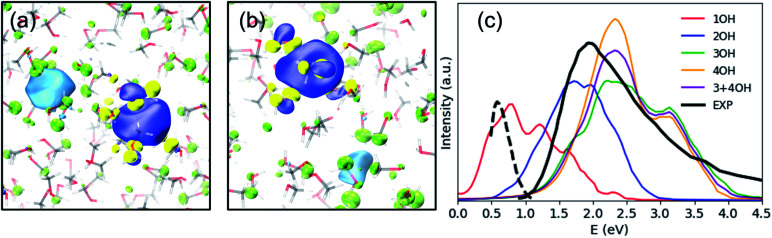
SOMO (blue – positive values, yellow – negative values) and the dominant virtual spin–orbital in the first excited state (light blue – positive values, green – negative values) for a typical (a) 2OH-cavity structure and (b) 4OH-cavity structure (shallow-trap ground state). Isovalue – 0.02 a.u. (c) Simulated and experimental electronic spectra of the solvated electron in methanol, including 20 excited states. The black solid line is an absorption spectrum of e_met_^−^ at thermal equilibrium,^21^ and the black broken line is of an early transient species observed in photoionization of liquid methanol.^61^

Previously, the one-electron model for e_met_^−^ assigned the photoabsorption maximum at 2.0 eV to three overlapping transitions from s- to p-type orbitals, similar to the case of e_aq_^−^, but the model did not reproduce the strong absorption by e_met_^−^ beyond 3.0 eV.^25^ On the other hand, our results suggest that the main absorption peak associated with e_met_^−^ in OH-cavities arises from electronic transitions to charge transfer states rather than the hydrogenic electronic transitions discussed so far; in fact, our calculations identified no hydrogenic transitions in the studied energy range. Our calculations predicted the absorption maximum to be *ca.* 2.2 eV for 3OH- and 4OH-cavities and *ca.* 1.7 eV for 2OH-cavities (see Fig. 3(c)). With a moderate blue shift of 0.2 eV, these values agree reasonably well with the experimentally observed absorption maxima for the shallow (1.5 eV) and the deep trap (2.0 eV) states.^12^ We ensured that the charge-transfer excited states are not spurious by performing the Mulliken averaged configuration diagnostic^58–60^ as described in the ESI, (Section D).†

Photoabsorption by e_met_^−^ in 1OH-cavities is weak and exhibits an absorption maximum below 1 eV. Nevertheless, its band closely resembles the photoabsorption spectrum (broken line in Fig. 3(c)) of an intermediate species reported by Thaller *et al.*^61^ The relatively strong photoabsorption extending beyond 3.0 eV is also ascribed to charge-transfer transitions, which explains why the spectral features in this energy region are different from those for e_aq_^−^.

What causes this difference in the nature of the excited states for e_met_^−^ between the previous and current computations? We believe that the one-electron model calculations confined the excess electron to a single cavity too strongly and prevented charge transfer to nearby cavities. To examine this problem, we performed embedded TDDFT calculations for one of the four OH-cavity structures, in which the methanol molecules forming the 4OH-cavity were embedded in the effective potential confining the electron in the cavity. As anticipated, the calculation provided only transitions from s- to p-type orbitals (after removal of spurious delocalized states), as described in more detail in the ESI (Section E).†

Charge transfer to spatial voids has previously been found for photoabsorption by e_sol_^−^ in tetrahydrofuran (THF),^62^ although the first three excited states are still localized in the central cavity with a diminished hydrogenic character. In this sense, e_sol_^−^ in THF exhibits an intermediate character between e_met_^−^ and e_aq_^−^. This correlates well with the densities (water: 998, THF: 890, methanol: 793 kg m^−3^ at 293 K) and the occurrence of voids in these liquids.

Since the excited states of e_met_^−^ have a charge-transfer character, the internal conversion of e_met_^−^ can be viewed as reverse non-radiative charge transfer. If this picture also holds for other primary alcohols, the rate of internal conversion possibly varies with the carbon chain length, since this changes the distance between the two cavities: the longer the alkyl group, the larger the lifetime of the excited state. Even though the two cavities may not be located at opposite ends of the alcohol molecule, the distance between the two cavities is correlated with the chain length. This agrees with the measured lifetime for the excited states of e_sol_^−^ in simple alcohols, which tends to increase with increasing carbon chain length.^23,27,28,63^

**Fig. 4:**
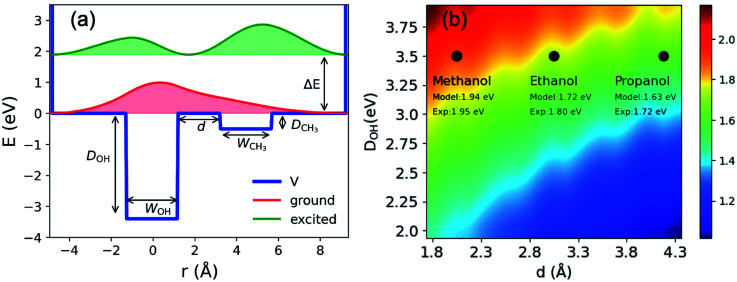
(a) Double square well model: potential; energies and probability density distributions of the ground and first excited states. *W*_OH_ and *W*_CH_3__ are width of the two wells; *d* is the distance between the wells; *D*_OH_; *D*_CH_3__ are the well depths and Δ*E* is the excitation energy between the ground and the first excited states. (b) Excitation energies (eV) obtained from the double square well model with different inter-cavity distances *d* and well depths *D*_OH_. The other parameters are: *D*_CH_3__ eV, *W*_OH_ = 2.2 Å and *W*_CH_3__ = 2.5 Å. The experimental values indicated in the figure are taken from ref. 21 and 63 and the references therein.

To illustrate charge-transfer excitation of e_met_^−^, a simple one-dimensional double square-well model is considered. Here we assume the well depth to be equal to that for the *n*OH- (ground) and CH_3_- (excited) cavities, its width to be the electron gyration radius, and the distance between the two wells to be the alkyl chain length (see Fig. 4(a)). Using appropriate values for these parameters, this simple model produces reasonably good agreement between the calculated excitation energies to the first excited states and the experimental spectra (see Fig. 4(b) and ESI, Section H†). The wave functions are mostly localized in the corresponding wells (see Fig. 4(a)). Increasing the distance between the wells, we obtain a red-shifted absorption peak maximum as experimentally observed for e_sol_^−^ in ethanol and propanol. A more precise model can be constructed by assuming three-dimensional multiple shallow wells around the central deep well; however, here we restricted ourselves to the simplest double square-well model to illustrate the essence of the physics involved.

## 4 Conclusions

By performing the first condensed-phase MD simulation with high-level electronic structure theory for excess electrons in methanol, we identified the bound states of the solvated electrons and classified them according to the number of OH- (and CH_3_-) groups contributing to the cavity. Upon electron injection, the excess electrons occupy naturally preexisting trapping sites formed by CH_3_-groups and then flow to more energetically stable OH-sites. Dangling OH-bonds are stochastically formed by hydrogen-bonding dynamics in solution. This trap-seeking behavior of the excess electrons changes to a trap-digging one once the excess electrons are trapped in the OH-sites, and the four methanol molecules in the first solvation shell reorient themselves one by one to coordinate the OH-groups to the electron. Thus, there are four trap states classified by the number of coordinated hydroxy groups rather than the two (shallow and deep) that are suggested by the experiments. The computed VBEs for these four trap states agree with the range of VBE values experimentally measured. Another important conclusion of the present study is a difference in the nature of the excited electronic states for e_met_^−^ from those for e_aq_^−^: the former are charge-transfer states, and the latter are hydrogenic ones. The charge-transfer nature explains the intense photoabsorption profile for e_met_^−^ beyond 3.0 eV, which is not present in the spectrum of e_aq_^−^, and the internal conversion time for e_sol_^−^ that varies with the alkyl chain length. Electronic transitions of e_met_^−^ are rationalized with a simple asymmetric double square-well potential; the model provides qualitative insights into the spectroscopy of e_sol_^−^ in other primary alcohols, explaining the alkyl-chain-length dependence of the maximum absorption peak and the internal conversion time. These findings may be exploited for the rational design of new reduction reagents in alcohol solutions.

## Data availability

Experimental and computational data are available from the authors upon a reasonable request.

## Author contributions

TS and VR conceived the project. JL and VR performed theoretical simulations, and YY and TS analyzed experimental results. All authors have contributed to the discussion and writing of the paper.

## Conflicts of interest

There are no conflicts to declare.

## Supplementary Material

SC-013-D1SC06666H-s001
