# Supplementary material for: Shallow and deep trap states of solvated electrons in methanol and their formation, electronic excitation, and relaxation dynamics
Source: Chem Sci. 2022 Mar 11;13(13):3837–44. doi: 10.1039/d1sc06666h (PMC8966712; doi:10.1039/d1sc06666h)
Supplement: SC-013-D1SC06666H-s001 [file SC-013-D1SC06666H-s001.pdf]

Electronic Supplementary Material (ESI) for Chemical Science.
This journal is © The Royal Society of Chemistry 2022

Supplementary Materials for Shallow and deep trap states of solvated electrons in methanol and their formation, electronic excitation, and relaxation dynamics

Jinggang Lan,[1,2,*] Yo-ichi Yamamoto,[3] Toshinori Suzuki,[3,†] and Vladimir V. Rybkin[1,4,‡]

[1]*Department of Chemistry, University of Zurich, Winterthurerstrasse 190, Zurich 8057, Switzerland*

[2]*Current address: Chaire de Simulation al'Echelle Atomique (CSEA), Ecole Polytechnique Fédérale de Lausanne (EPFL), CH-1015 Lausanne, Switzerland;*

[3]*Department of Chemistry, Graduate School of Science, Kyoto University, Kyoto 606-8502, Japan*

[4]*Current address: HQS Quantum Simulations GmbH, Haid-und-Neu-Straße 7, D-76131 Karlsruhe, Germany*

(Dated: February 21, 2022)

* jinggang.lan@epfl.ch

† suzuki@kuchem.kyoto-u.ac.jp

‡ vladimir.rybkin@quantumsimulations.de

A. Hybrid-functional calculations

The hybrid density functional based molecular dynamics simulations were carried out using PBE(α) with 50%-HFX (see Eq.1) with non-local van der Waals rVV10 correction [1], which has been proved to give an accurate description of the aqueous excess electron [2] and is similar to the one applied by the group of Pasquarello [3, 4]. Molecular orbitals of the valence electrons were expanded in the TZV2P basis sets[5], while atomic core electrons were described through Goedecker-Teter-Hutter (GTH) pseudopotentials corresponding to the PBE functional [6, 7]. Exact exchange integrals were calculated within the auxiliary density matrix method (ADMM) approximation [8]. In addition, the truncated Coulomb operator [9] has been applied for the exchange calculations with the cutoff radius approximately equal to half the length of the smallest edge of the simulation cell, together with the Schwarz integral screening with the threshold of 10^{-10} a. u. The cutoff for the auxiliary plane waves was 800 Ry for both system sizes.

$$E_{\mathrm{xc}}^{\mathrm{PBEh}(\alpha)} = \frac{1}{2}E_{\mathrm{x}}^{\mathrm{HF}} + \frac{1}{2}E_{\mathrm{x}}^{\mathrm{PBEh}} + E_{\mathrm{c}}^{\mathrm{PBEh}} \tag{1}$$

B. MP2 calculations

The MP2 correlation energies and forces were computed within the resolution-of-identity approximation in the Gaussian and plane waves framework [10, 11] with triple-zeta quality correlation-consistent basis set [12]. The truncated Coulomb operator [9] was applied for the exchange integrals calculations with the cutoff radius approximately equal to half the length of the smallest edge of the simulation cell, 6.8 Å, together with the Schwarz integral screening with the threshold of 10^{-10} a. u. self-consistent field convergence criterion was set to $5 \cdot 10^{-7}$ a.u. The plane waves cutoff for the Hartree-Fock part of the calculations was 500 Ry, whereas the cutoff for the correlation energy calculations was 300 Ry. Atomic core electrons were described through Goedecker-Teter-Hutter (GTH) pseudopotentials [6, 7] optimized for the Hartree-Fock theory. For this system size, each of the energy and force calculation on 512 Cray XC50 hybrid CPU/GPU compute nodes took ca. 30 minutes of wall-time.

C. Molecular dynamics

The initial conditions for trajectories of solvated electron were prepared by equilibrating liquid methanol in an NVT ensemble at 300 K using the canonical Langevin thermostat [13]. The cell sizes correspond to the experimental densities of liquid methanol and are: $13.9 \times 13.9 \times 13.9$ Å for the smaller system (39 molecules) and $15.35 \times 15.35 \times 15.35$ Å for the larger system (55 molecules). Production trajectories have been integrated in an NVE ensemble. For all hybrid DFT MD simulations, a time-step of 0.5 fs was used. The MP2-based molecular dynamics employed a multiple time-step integrator, where the fast time step (0.25 fs) corresponds to a PBE(α) with 50%-HFX simulation, while the slow time step (1.5 fs) is based on MP2.

D. Electronic spectra

1. Time-dependent DFT calculations

We have computed electronic spectra using time-dependent density functional perturbation theory (TDDFPT), often referred to as time-dependent DFT (TDDFT). We used PBE(α) with 50%-HFX within the ADMM approximation for the exact exchange [8] and a triple-zeta quality basis set GTH-TZV2P [5]. We used 67 snapshots for 2 OH-, 3 OH-, 4 OH-cavities and 20 snapshots for 1 OH-cavities.

We have computed 20 excited states for each of the frames selected from the MD trajectories of the larger system.

2. Spurious charge-transfer state diagnostics

We apply the Mulliken averaged configuration (M_{AC}) spurious charge-transfer (CT) state diagnostic to assess physical relevance of electronic spectra computed here with a hybrid functional.

M_{AC} is a lower estimate of the CT transition energy [14]. Therefore, TDDFT excitation energies, E_{TDDFT}, below this value are regarded as spurious:

$$E_{TDDFT} < M_{AC} : \text{spurious state} \tag{2}$$

$$E_{TDDFT} > M_{AC} : \text{real state} \tag{3}$$

The original M_{AC} index is computed as follows:

$$M_{AC} = IP_D - EA_A - \frac{1}{R}, \tag{4}$$

where IP_D is the ionization potential of the donor state, EA_A is the electron affinity of the acceptor state, and R is the distance between charge distribution centres of electron donor and acceptor states.

In the context of this work, IP_D are VBEs of the ground states, *i.e.* nOH-cavities. The acceptor state is the CH_3-cavity with $EA_A = VBE$. The parameter values determined in this work are: $IP_D(1OH) = 1.8$, $IP_D(2OH) = 2.3$, $IP_D(3OH) = 2.6$, $IP_D(1OH) = 3.2$, and $EA_A = 0.4$ eV. Since M_{AC} increases with charge separation R we computed the upper bound of the index by taking the maximum possible separation equal to the half of the cell simulation cell length: 7.5 Å. The resulting M_{AC} for di erent cavity types are shown in Figure 1. Even at the maximum possible donor/acceptor state separation, M_{AC} is lower than computed absorption maximum for all cavity types. Note that for most structures the separations are ca. 5-6 Å(see the justification in **Section H. Double square well model**).

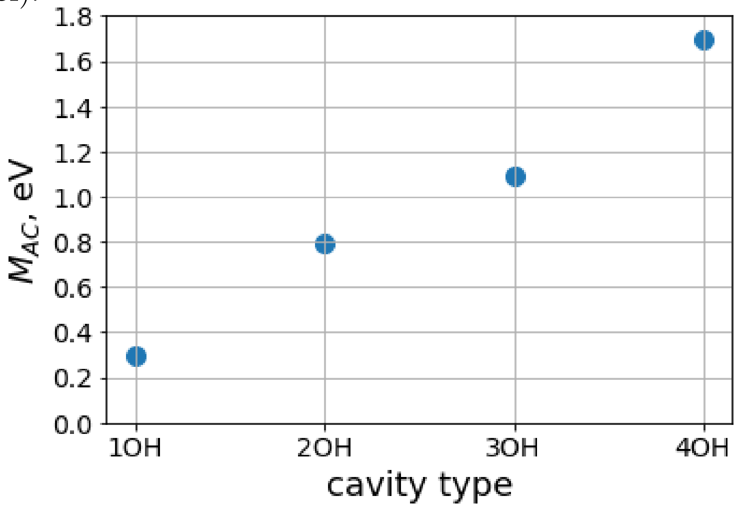

Figure 1: Upper bound of the M_{AC} index for different cavity structures computed using binding energies.

We have also computed the elaborate version [15, 16] of the index based on structure-specific Kohn-Sham quantities:

$$M_{AC} = \frac{\sum_{ia} c_{ia}^2(\epsilon_a - \epsilon_i)}{\sum_{ia} c_{ia}^2} - \frac{1}{R}, \tag{5}$$

where i and a are indices of occupied and virtual orbitals, ϵ_i, ϵ_a are the corresponding Kohn-Sham orbital energies, c_{ia} are excitation amplitudes, and R is the distance between charge distribution centres of electron donor and acceptor states. We restricted ourselves to the two lowest excited states of a representative 4 OH-cavity structure (see Table I). TDDFT excitation energies are significantly higher than the index, which is a clear evidence of their physical (non-spurious) nature.

Table I: M_{AC} index and TDDFT excitation energies for a representative *4OH*-cavity structure.

state	M_{AC}, eV	E_{TDDFT}, eV
1	0.94	2.15
2	0.35	2.34

E. Embedded calculations

Embedded calculations have been undertaken to prove the effect of confinement on the nature of the excited states. We have applied density functional embedding theory [17] based on the unique embedding potential as implemented in CP2K [18]. As a proof-of-the-concept, the calculations have been performed on one representative 4 OH-cavity structure with 59 methanol molecules. Four methanol molecules forming the cavity and an extra electron have been selected as an embedded cluster, whereas the rest served as an environment. PBE functional was used to describe the environment and interaction between the subsystems, whereas PBE(α) with 50%-HFX was chosen as a high-level method applied to the embedded cluster. For the embedded cluster, an electronic spectrum was computed using time-dependent DFT. The virtual orbitals, responsible for the most absorption of the embedded cluster, are similar to the hydrogenic ones of the solvated electron in water (see Figures 2, 3, 4).

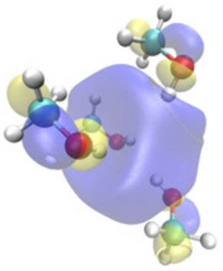

Figure 2: s-type orbital of the embedded cluster (ground state).

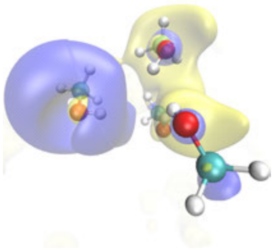

Figure 3: p-type orbital of the embedded cluster (first excited state).

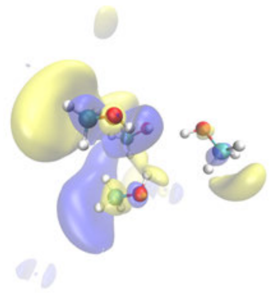

Figure 4: d-type orbital of the embedded cluster.

F. Volumetric data analysis and processing: spin density gyration radius and shape anisotropy

Since the position operator $\mathbf{r}$ is not defined under periodic boundary conditions the gyration radius is not defined either. However, the simulation periodic cell is large enough to allow the treatment of periodic spin as non-periodic. For this purpose, the the Gaussian cube files, used to store spin density distributions, have been centered so that the spin density decays to zero at the boundaries of the cell.

The centre of spin density distribution $\rho^s(\mathbf{r})$ is given as follows:

$$\mathbf{r}_c = \int \rho^s(\mathbf{r})\mathbf{r}d\mathbf{r} \tag{6}$$

The second moment tensor reads:

$$\mathbf{S} = \int (\mathbf{r} - \mathbf{r}_c)(\mathbf{r} - \mathbf{r}_c)\rho^s(\mathbf{r})d\mathbf{r} \tag{7}$$

Then the gyration radius is calculated as:

$$r_g = \sqrt{\lambda_1^2 + \lambda_2^2 + \lambda_3^2}, \tag{8}$$

where λ_1, λ_2, λ_3 are the eigenvalues of $\mathbf{S}$.

With the spin density distribution on the real-space grid:

$$\mathbf{r}_c = \sum_{i=N}^{N} \rho^s(\mathbf{r})\mathbf{r}, \tag{9}$$

the elements of **S** are calculated as follows:

$$S_{xx} = \sum_{i=1}^{N} [(y_i - y_c)^2 + (z_i - z_c)^2] \rho^s(\mathbf{r}_i), \tag{10}$$

$$S_{yy} = \sum_{i=1}^{N} [(x_i - x_c)^2 + (x_i - x_c)^2] \rho^s(\mathbf{r}_i), \tag{11}$$

$$S_{zz} = \sum_{i=1}^{N} [(x_i - x_c)^2 + (y_i - y_c)^2] \rho^s(\mathbf{r}_i), \tag{12}$$

$$S_{xy} = -\sum_{i=1}^{N} [(x_i - x_c)^2 + (y_i - y_c)^2] \rho^s(\mathbf{r}_i), \tag{13}$$

$$S_{xz} = -\sum_{i=1}^{N} [(x_i - x_c)^2 + (z_i - z_c)^2] \rho^s(\mathbf{r}_i), \tag{14}$$

$$S_{yz} = -\sum_{i=1}^{N} [(y_i - y_c)^2 + (z_i - z_c)^2] \rho^s(\mathbf{r}_i), \tag{15}$$

where the summation runs over the N grid points.

Shape anisotropy is calculated as follows:

$$\kappa^2 = \frac{3}{2} \frac{\lambda_x^4 + \lambda_y^4 + \lambda_z^4}{(\lambda_x^2 + \lambda_y^2 + \lambda_z^2)^2} - \frac{1}{2} \tag{16}$$

G. Vertical electron binding energies (VBE), eV

VBE were computed as described in the Methods section of the main text. The diagram of VBE calculations is shown in Fig.5

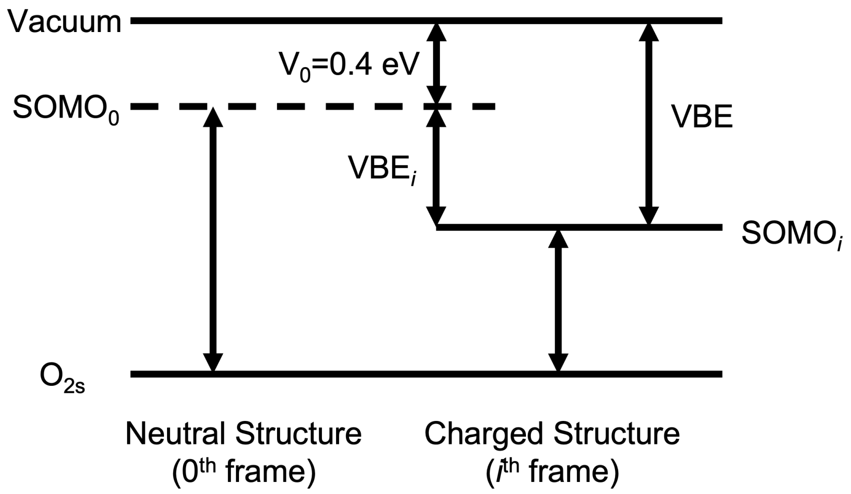

Figure 5: Energy diagram of calculating vertical electron binding energy based on Eq. 1

The V_0 is estimated against the experiment. It is also possible to estimate based on *ab initio* calculations. To this end, we chose the structure of the neat methanol and cut a 41 molecules cluster around the CH_3-cavity. The binding energy in the non-periodic system is well-defined, which is the energy level of the SOMO. The calculations of the cluster were carried out using ORCA code.[19] We adopted the PBEh(50%) functional with different basis sets: TZVP, TZVPP, def2-TZVP and def-TZVPP. The vertical binding energies (VBE $\approx$ -E_{SOMO}) are listed in Table I. The VBE of the neat methanol is ca. 0.6 eV, which agrees with the assumed value V_0 = 0.4 eV.

Table II: Vertical binding energies of the cluster as obtained from PBEh(50%) with different basis sets

	TZVP	TZVPP	def2-TZVP	def2-TZVPP
VBE (eV)	0.61	0.65	0.64	0.65

We have undertaken an attempt to extrapolate the VBEs to the infinite simulation cell size using two data points for each cavity type corresponding to the 39- and 55-molecule

Table III: Vertical binding energies for the trap states obtained from periodic calculations. Column **39 molecules, MP2** corresponds the values calculated for the frames extracted from the MP2 trajectories. Values in the table were plotted in Figure 2 of the main text.

structure	39 molecules	39 molecules, MP2	55 molecules
1OH	1.0 ± 0.3	-	1.4 ± 0.3
2OH	2.0 ± 0.3	1.4 ± 0.3	1.9 ± 0.2
3OH	2.6 ± 0.4	2.1 ± 0.2	2.2 ± 0.3
4OH	2.6 ± 0.4	2.7 ± 0.3	2.8 ± 0.3

systems as shown in Figure 6. The extrapolated values are unphysical, which is explained by the close VBEs for both systems and large error margins. In fact, within the error bars shown in Table III the VBEs for the two system sizes are indistinguishable.

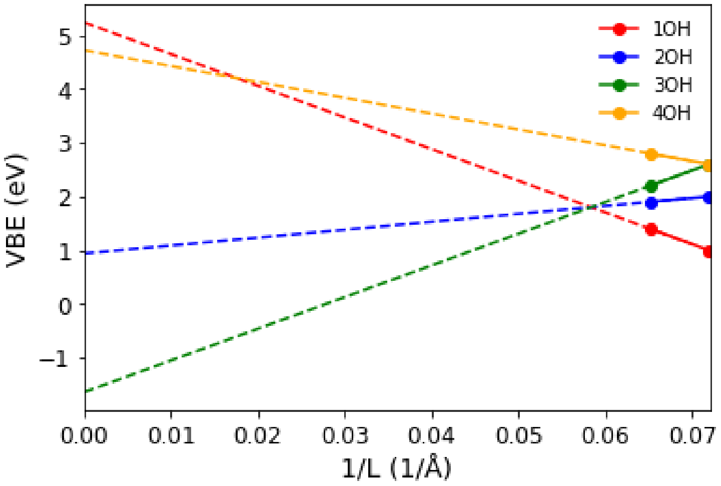

Figure 6: VBE extrapolation to infinite cell size. L is the cell length; two data points correspond to the 39- and 55-molecule simulation cells.

H. Double square well model

Table IV: Double square well model: parameters and results. W_{OH} and W_{R} (R is the corresponding alkyl radical: methyl, ethyl, propyl) are width of the two wells, corresponding to the gyration radii of the states; d is the distance between the wells, correlated with carbon backbone length; D_{OH} and D_{R} are the well depths corresponding to the electron binding energies. ΔE is the energy gap between the ground and the first excited states. Notation is clarified in Figure 4

	methanol	ethanol	propanol
W_{OH}(Å)	2.2	2.2	2.2
W_{R} (Å)	2.5	2.5	2.5
d(Å)	2.0	3.0	4.0
D_{OH}(eV)	3.4	3.4	3.4
$D_{\mathrm{CH_3}}/D_{\mathrm{C_2H_5}}$ (eV)	0.5	0.5	0.5
ΔE (eV)	1.94	1.80	1.72

[1] R. Sabatini, T. Gorni, and S. de Gironcoli, Phys. Rev. B **87**, 041108 (2013).

[2] J. Wilhelm, J. VandeVondele, and V. V. Rybkin, Angewandte Chemie International Edition **58**, 3890 (2019).

[3] M. Pizzochero, F. Ambrosio, and A. Pasquarello, Chemical Science (2019).

[4] F. Ambrosio, G. Miceli, and A. Pasquarello, The Journal of Physical Chemistry Letters **8**, 2055 (2017).

[5] J. VandeVondele and J. Hutter, The Journal of Chemical Physics **127**, 114105 (2007).

[6] S. Goedecker, M. Teter, and J. Hutter, Physical Review B **54**, 1703 (1996).

[7] C. Hartwigsen, S. Gœdecker, and J. Hutter, Physical Review B **58**, 3641 (1998).

[8] M. Guidon, J. Hutter, and J. VandeVondele, Journal of Chemical Theory and Computation **6**, 2348 (2010).

[9] M. Guidon, J. Hutter, and J. VandeVondele, Journal of Chemical Theory and Computation **5**, 3010 (2009).

[10] M. Del Ben, J. Hutter, and J. VandeVondele, The Journal of Chemical Physics **143**, 102803 (2015).

[11] V. V. Rybkin and J. VandeVondele, Journal of Chemical Theory and Computation **12**, 2214 (2016).

[12] M. Del Ben, J. Hutter, and J. VandeVondele, Journal of Chemical Theory and Computation **9**, 2654 (2013).

[13] M. Ceriotti, G. Bussi, and M. Parrinello, Phys. Rev. Lett. **102**, 020601 (2009).

[14] R. S. Mulliken, Journal of the American Chemical Society **74**, 811 (1952), https://doi.org/10.1021/ja01123a067.

[15] M. Campetella, F. Maschietto, M. J. Frisch, G. Scalmani, I. Ciofini, and C. Adamo, Journal of Computational Chemistry **38**, 2151 (2017), https://onlinelibrary.wiley.com/doi/pdf/10.1002/jcc.24862.

[16] F. Maschietto, M. Campetella, J. Sanz García, C. Adamo, and I. Ciofini, The Journal of Chemical Physics **154**, 204102 (2021), https://doi.org/10.1063/5.0050680.

[17] C. Huang, M. Pavone, and E. A. Carter, The Journal of Chemical Physics **134**, 154110 (2011), https://doi.org/10.1063/1.3577516.

[18] T. D. Kühne, M. Iannuzzi, M. Del Ben, V. V. Rybkin, P. Seewald, F. Stein, T. Laino, R. Z. Khaliullin, O. Schütt, F. Schiffmann, D. Golze, J. Wilhelm, S. Chulkov, M. H. Bani-Hashemian, V. Weber, U. Borštnik, M. Taillefumier, A. S. Jakobovits, A. Lazzaro, H. Pabst, T. Müller, R. Schade, M. Guidon, S. Andermatt, N. Holmberg, G. K. Schenter, A. Hehn, A. Bussy, F. Belleflamme, G. Tabacchi, A. Glöß, M. Lass, I. Bethune, C. J. Mundy, C. Plessl, M. Watkins, J. VandeVondele, M. Krack, and J. Hutter, The Journal of Chemical Physics **152**, 194103 (2020).

[19] F. Neese, Wiley Interdisciplinary Reviews: Computational Molecular Science **2**, 73 (2012).
